# Topical Omidenepag Isopropyl Lowers Intraocular Pressure in Normal and Glaucomatous Cats: A Pilot Study

**DOI:** 10.1111/vop.70125

**Published:** 2025-12-03

**Authors:** Kazuya Oikawa, Julie A. Kiland, Hayden G. Zaluckyj, Anjali Rai, Virginia Mathu, Gillian J. McLellan

**Affiliations:** ^1^ Surgical Sciences University of Wisconsin‐Madison Madison Wisconsin USA; ^2^ Ophthalmology and Visual Sciences University of Wisconsin‐Madison Madison Wisconsin USA; ^3^ McPherson Eye Research Institute Madison Wisconsin USA

**Keywords:** cats, EP2 agonist, glaucoma, intraocular pressure, Omidenepag isopropyl

## Abstract

**Purpose:**

To evaluate the effects of topical prostaglandin E2 receptor 2 (EP2) agonist, omidenepag isopropyl (OMDI), on intraocular pressure (IOP) and pupil diameter (PD) in normal cats and cats with feline congenital glaucoma (FCG).

**Animals Studied:**

Ten FCG cats and 8 normal cats.

**Methods:**

In this prospective, randomized, placebo‐controlled, masked pilot study, normal and FCG cats received one drop of 0.002% OMDI ophthalmic solution in one eye and artificial tears in the contralateral eye. IOP and PD were measured by a masked observer at baseline and at multiple time points up to 32–36 h post‐administration.

**Results:**

Topical 0.002% OMDI significantly lowered IOP compared to controls in both normal and FCG cats. In normal cats, the effect was significant up to 12 h after instillation, with a maximal reduction from baseline of 7.3 mmHg (43.5%). In FCG cats, the effect was significant from 2 to 8 h after instillation, with a maximal reduction from baseline of 16.7 mmHg (60.2%). No significant effect on pupil diameter or signs of ocular irritation was observed in either group.

**Conclusions:**

A single topical drop of 0.002% OMDI significantly lowers IOP in normal and FCG cats without inducing miosis. Our findings support that topical selective EP2 agonists may be a promising therapeutic option for feline glaucoma and provide information relevant to dosing intervals for future studies.

## 1 Introduction

Glaucoma is an optic neuropathy that leads to irreversible blindness in many species, including cats. Intraocular pressure (IOP) remains the only treatable risk factor for this vision‐threatening disease. Feline glaucoma is often insidious in onset and gradually progressive with very subtle clinical signs, but it is a common cause of enucleation in feline patients [1]. While secondary glaucoma is the more prevalent form, several primary glaucomas have been recognized in cats, including feline congenital glaucoma (FCG) and feline open angle glaucoma (FOAG) [2]. An autosomal recessive form of FCG caused by a mutation in *LTBP2* [3] has been studied extensively as a valuable translational research model [4] and has been used in pharmacological studies in cats to evaluate a number of commercially available anti‐glaucoma medications [5, 6].

Feline glaucoma represents a therapeutic challenge for veterinary clinicians as few available drugs are both well‐tolerated and clinically effective at consistently reducing IOP in cats. Thus, there is a critical need for a new therapeutic approach for feline glaucoma. In human and canine glaucomas, prostaglandin F (FP) receptor agonists such as latanoprost are considered highly effective and represent a mainstay of treatment. However, these agents lack a consistent and sustained IOP‐lowering effect in cats [6], likely due to species differences in FP receptor expression in the anterior segment. In contrast to FP receptor agonists, previous research studies have shown that topical application of prostaglandin E2 (PGE2), a non‐specific prostaglandin E (EP) receptor agonist, results in a significant reduction in IOP in normal feline eyes with no overt miosis [7].

Omidenepag isopropyl (OMDI) is a first‐in‐class topical ophthalmic anti‐glaucoma medication. OMDI has been available in Japan since 2018 (Eybelis) and was FDA‐approved in the USA in 2022 (Omlonti) for treatment of glaucoma and ocular hypertension in human patients, with once daily administration [8]. Following topical administration, OMDI is hydrolyzed by endogenous corneal esterases into its active free acid form, omidenepag (OMD). OMD is then released in the aqueous humor and acts as a non‐prostaglandin, highly selective prostaglandin E receptor EP2 subtype (EP2 receptor) agonist, promoting aqueous outflow. Notably, the EP2 receptor is expressed in many ocular tissues, such as the ciliary body, across different species, including cats [9]. A recent case series highlighted a potential IOP‐lowering effect of 0.002% OMDI ophthalmic solution in feline glaucoma patients that were not responsive to conventional anti‐glaucoma medications [10]. Another study demonstrated that topical OMDI may reduce IOP for at least 4 h post‐instillation in normal feline eyes [11]. Prior reports have indicated that topical OMDI does not induce overt miosis in client‐owned cats [10, 11]. Despite its potential promise for the treatment of feline glaucoma, no published studies have rigorously evaluated ocular hypotensive effects or effects on pupil diameter (PD) of OMDI in normal cats and cats with spontaneous glaucoma in a controlled experimental setting. This pilot study aimed to determine the effects of 0.002% OMDI ophthalmic solution on IOP and PD in normal and FCG cats.

## 2 Materials and Methods

### 2.1 Ethics Statement

This study complies with the Guidelines for Ethical Research in Veterinary Ophthalmology (GERVO). All animal procedures were conducted in accordance with the Association for Research in Vision Science and Ophthalmology (ARVO) Statement on the Use of Animals in Ophthalmic and Vision Research, the NIH Guide for the Care and Use of Laboratory Animals, and with the approval of the University of Wisconsin‐Madison Institutional Animal Care and Use Committee.

### 2.2 Animals

Ten cats homozygous for the *LTBP2* mutation causal for FCG (mean age ± SD = 0.7 ± 0.21 years; 5 males, 5 females) and 8 normal cats (0.68 ± 0.06 years; 2 males, 6 females) were used for this study. All animals were derived from a research breeding colony and were group‐housed in an animal care facility, under standard, controlled environmental conditions, with a 12 h light–dark cycle. All animals were phenotyped by ophthalmic examination including slit‐lamp biomicroscopy (SL‐17; Kowa Company Ltd., Tokyo, Japan) and tonometry (TonoVet; Icare Finland Oy, Helsinki, Finland) performed by a board‐certified veterinary ophthalmologist (GJM) to ensure freedom from confounding ocular disease other than glaucoma. All animals included in the study were well‐acclimated to IOP measurements via routine weekly tonometry from early in life by experienced lab personnel.

### 2.3 Study Design

This study was a prospective, randomized, placebo‐controlled masked study. Rebound tonometry was performed as previously validated in normal and FCG cats [12], with three tonometer readings averaged to provide a single value for each cat at each time point. Horizontal PD was measured using digital calipers (Traceable Digital Carbon Fiber Calipers; Fisher Scientific, Waltham, MA, USA) as previously described [5]. Pre‐treatment, baseline measurements of IOP and PD were recorded for both eyes of all cats at 2‐h intervals from 8 AM to 8 PM prior to the initiation of the treatment phase. On the treatment day, a single drop (~30 μL) of 0.002% OMDI ophthalmic solution (Eybelis; Santen Pharmaceutical Co. Ltd., Osaka, Japan) was administered topically to one randomly assigned eye of each cat immediately following the 8 AM measurements. The contralateral eye received a single drop of artificial tears (Refresh Tears; AbbVie, IL, USA) to serve as a control. Following a single topical treatment, IOP and PD measurements were obtained for both eyes of all cats every 2 h for the first 0–12 h and then at 24 h post treatment. Additional IOP and PD measurements were obtained for normal cats (at 2‐h intervals from 24 h to 32 h post instillation) and for glaucomatous cats (at 6‐h intervals from 24 h to 36 h post instillation). In the second cohort studied (normal cats), the measurement interval was modified to capture time points of greatest interest. The single observer responsible for all IOP and PD measurements was masked to the treatment status of each eye.

### 2.4 Ocular Irritation Assessment

Tolerability of the topical treatment was assessed immediately following administration and at each subsequent measurement time point. Signs of ocular irritation, including blepharospasm, conjunctival hyperemia, chemosis, and ocular discharge, were monitored using a well‐described scale [5].

### 2.5 Statistics

Statistical analyses were performed using Prism 10 (ver. 10.4.1; GraphPad, San Diego, CA). Data normality was assessed by the Shapiro–Wilk test and Kolmogorov–Smirnov test. IOP and PD values between OMDI‐treated eyes and contralateral control eyes at each time point were compared using a two‐way repeated‐measures ANOVA or mixed‐effects model with Holm‐Šídák's multiple comparisons post hoc test. Within each group (normal and FCG), measurement values at each time point were compared to baseline values for the same eye using repeated measures ANOVA with Holm‐Šídák's multiple comparisons post hoc testing where appropriate. For all analyses, *p* ≤ 0.05 was considered statistically significant.

## 3 Results

### 3.1 Effects of 0.002% OMDI Ophthalmic Solution on IOP in Normal Cats

**FIGURE 1:**
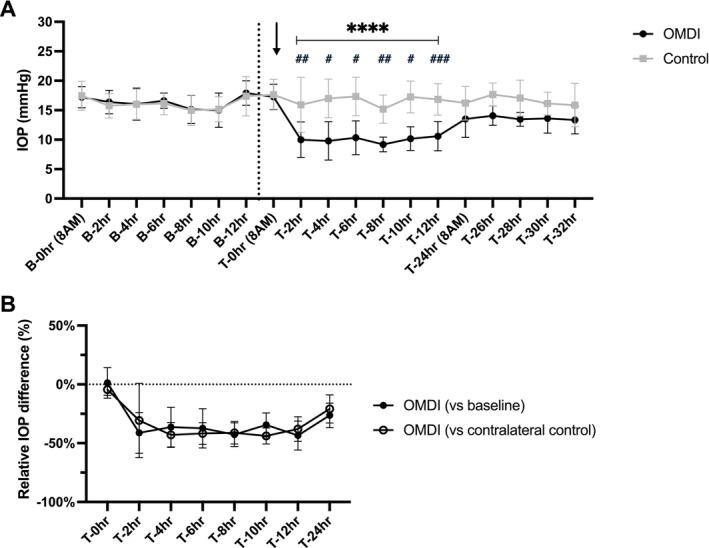
Effects of a single topical administration of 0.002% OMDI ophthalmic solution on IOP in eight normal cats. Plots show: (A) mean IOP values of normal feline eyes pre‐treatment, baseline and post‐treatment with either 0.002% OMDI ophthalmic solution or placebo‐control (artificial tears) are plotted. Arrow indicates the timing of topical drug administration. The dashed black line indicates the end of the pre‐treatment baseline phase. Error bars represent standard deviation. ^#^ *p* < 0.05, ^##^ *p* < 0.01, ^###^ *p* < 0.001 relative to pre‐treatment baseline; ****p < 0.0001 relative to contralateral placebo‐control (Holm‐Šídák's multiple comparisons post hoc test). (B) Mean percentage difference of IOP in OMDI‐treated eyes in normal cats relative to pretreatment baseline and contralateral control. Error bars represent 95% confidence interval.

The mean (±SD) IOP of normal feline eyes pre‐ and post‐treatment with either 0.002% OMDI ophthalmic solution or artificial tears is presented in Figure 1A. During the baseline period (from B‐0 to B‐12 h) and immediately prior to drug administration (T‐0 h), there was no significant difference in IOP between the eyes designated for OMDI and contralateral control eyes. Following a single administration, IOP values in OMDI‐treated eyes were significantly lower from 2 to 12 h post‐treatment compared with pre‐treatment baseline values in the same eyes (*p* < 0.05) and with the contralateral control eye values at the same time point (*p* < 0.0001). Within this period, OMDI‐treated eyes had a maximal IOP reduction of 7.3 ± 2.4 mmHg at 2 h post‐treatment (43.5% reduction; 95% CI, 31.2–55.8) and 7.2 ± 2.3 mmHg at 4 h post‐treatment (43.0% reduction; 95% CI, 32.4–53.6), relative to pre‐treatment baseline and contralateral controls, respectively (Figure 1B).

### 3.2 Effects of 0.002% OMDI Ophthalmic Solution on IOP in Glaucomatous Cats

**FIGURE 2:**
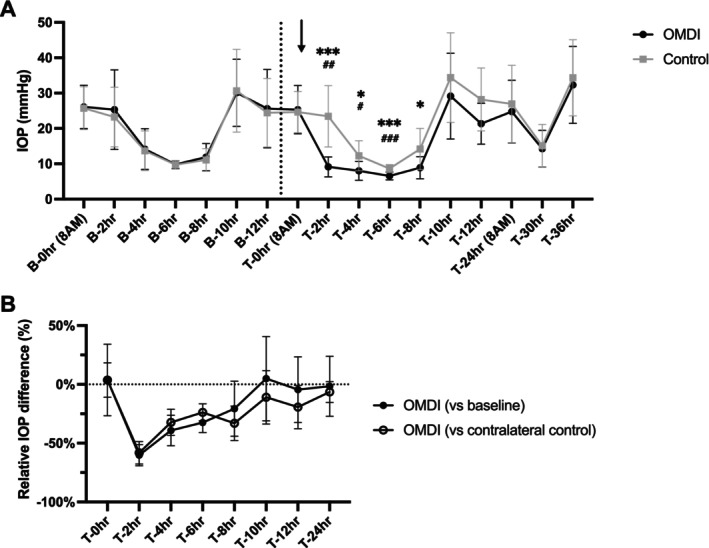
Effects of a single topical administration of 0.002% OMDI ophthalmic solution on IOP in ten glaucomatous cats. Plots show: (A) IOP values of eyes with feline congenital glaucoma pre‐treatment, baseline and post‐treatment with either 0.002% OMDI ophthalmic solution or placebo‐control (artificial tears). Arrow indicates the timing of topical drug administration. The dashed black line indicates the end of the pre‐treatment baseline phase. Error bars represent standard deviation. ^#^ *p* < 0.05, ^##^ *p* < 0.01, ^###^ *p* < 0.001 relative to pre‐treatment baseline; *p < 0.05, *p < 0.001 relative to contralateral placebo‐control (Holm‐Šídák's multiple comparisons post hoc test). (B) Mean percentage difference of IOP in OMDI‐treated eyes in glaucomatous cats relative to pretreatment baseline and contralateral control. Error bars represent 95% confidence interval.

Mean (±SD) pre‐ and post‐treatment IOP values in OMDI‐treated and contralateral control eyes in FCG cats are presented in Figure 2A. There were no significant between‐eye differences in IOP at any time point of pre‐treatment baseline measurement phase and immediately prior to drug administration (T‐0 h; *p* > 0.52). During pre‐ and post‐treatment periods, there was marked circadian fluctuation in IOP in glaucomatous eyes, with the lowest IOP observed at 2 PM. In FCG cats, OMDI‐treated eyes had significantly lower IOP compared to contralateral control at 2–8 h (*p* < 0.037) and compared to pre‐treatment baseline at 2–6 h post‐instillation (*p* < 0.045). At 2 h post‐treatment, OMDI‐treated eyes had a maximal IOP reduction of 16.7 ± 10.6 mmHg (60.2% reduction; 95% CI, 51.2–69.2) relative to pre‐treatment baseline and 14.3 ± 6.9 mmHg (58.2% reduction; 95% CI, 48.7–67.7) relative to contralateral control (Figure 2B).

### 3.3 No Effects of Topical OMDI on PD in Normal and Glaucomatous Cats

Topical administration of 0.002% OMDI had no significant effect on PD in either normal or glaucomatous cats. No significant differences in PD between OMDI‐treated and contralateral control eyes were observed at any pre‐ or post‐treatment time points. Mean (±SD) maximal differences in PD between OMDI and control eyes following treatment were 0.11 ± 0.20 mm in normal cats and 0.31 ± 0.41 mm in FCG cats. These differences were not consistent, or statistically significant, and clinically negligible (*p* > 0.90).

### 3.4 No Major Adverse Effects Observed Following Topical Administration in Normal and Glaucomatous Cats

Throughout the study, no signs of ocular irritation, such as blepharospasm, conjunctival hyperemia, chemosis or ocular discharge were observed in any eyes of either normal or FCG cats.

## 4 Discussion

This pilot study, conducted under controlled experimental conditions, provides initial proof of concept and compelling evidence that a single topical administration of OMDI significantly lowers IOP in both normal and FCG cats. Our data also confirmed that this significant IOP‐lowering effect occurs without inducing miosis in either normal or glaucomatous eyes, providing an advantage over topical prostaglandin analogues in cats.

The maximal IOP reductions observed were 43.5% in normal cats and 60.2% in glaucomatous cats, which are considered robust and clinically meaningful. Our pilot study did not include a direct head‐to‐head comparison of IOP‐lowering effects between OMDI and other anti‐glaucoma medications. However, our results suggest that topical OMDI has an IOP‐lowering effect that compares favorably to that of other commercially available topical anti‐glaucoma medications studied under similar conditions in our lab [5, 6, 13, 14].

Significant IOP reduction was observed within 2 h following topical OMDI administration in normal and glaucomatous cats. This onset of action is consistent with that in previous studies of topical PGE2 agonists in cats [7]. This significant IOP‐lowering effect of topical OMDI was sustained for up to 12 h in normal cats, whereas in glaucomatous cats, this effect was only statistically significant for 6 to 8 h following instillation. The shorter duration of the IOP‐lowering effect in glaucomatous feline eyes may be attributable to their greater anterior chamber volume compared with normal eyes, which could lead to drug dilution thus reducing its effective duration. A previous case series reported BID‐QID dosing in cats with glaucoma, with dosing frequency selected at the clinician's discretion [10]. It is conceivable that cats may require more frequent dosing of 0.002% OMDI ophthalmic solution than the once‐daily regimen approved for treatment of human glaucoma, due to their larger anterior chamber size [15]. However, detailed pharmacokinetics and pharmacodynamic studies as well as determination of the most appropriate dosing frequency are beyond the scope of this short study and thus warrant further investigation. Notably, in this study, the timing of OMDI administration may also have affected the statistical sensitivity to detect significant differences in IOP between groups, as the efficacy of OMDI may have been masked by the marked circadian IOP fluctuations in both eyes that are characteristic of glaucomatous cats [13]. As shown in Figure 2A, glaucomatous cats generally exhibit lower IOP during the day and higher IOP at night. As topical OMDI was administered in the morning when IOP trends lower, a different dosing schedule and night‐time IOP measurements might reveal a more prolonged period of efficacy as observed in the normal feline cohort.

Clinical research on the use of OMDI in feline glaucoma has been extremely limited. While previous studies on topical PGE2 reported a substantial IOP reduction without causing miosis in normal cats [7], our results similarly demonstrated that topical OMDI has a minimal effect on PD in normal and glaucomatous feline eyes, consistent with previous reports in client‐owned animals [10, 11]. This lack of a miotic effect represents a key clinical advantage over conventional prostaglandin analogs [5, 6], as it enables longitudinal assessment of the retina and optic nerve head via fundoscopy and advanced imaging techniques, including optical coherence tomography, and would not appear to be contraindicated in eyes with anterior uveitis.

The main limitation of the current study was that the cohort of glaucomatous cats was affected by a single form of inherited primary congenital glaucoma. While this FCG cohort provided a relatively homogeneous group, this form of primary glaucoma is not necessarily representative of all glaucoma in the general feline population, in which secondary glaucoma is the most common [1]. Additionally, this study only evaluated the effect of a single dose. Thus, long‐term controlled studies are indicated to assess whether OMDI could be a viable treatment option for feline glaucoma.

One published case series has reported that topical 0.002% OMDI may effectively lower IOP in various forms of glaucoma in cats, including glaucoma secondary to uveitis; aqueous humor misdirection syndrome, and FOAG [10]. Cats with glaucoma unresponsive to a topical carbonic anhydrase inhibitor and beta‐blocker in that study showed variable responses to topical OMDI. The authors implied a possible association between disease severity and a lack of IOP‐lowering effect by topical OMDI. Ocular pathology in the proximal outflow pathway, including the trabecular meshwork, as well as the distal outflow pathway is likely to have increased with disease progression [16]. As OMDI is proposed to promote aqueous outflow via both conventional and uveoscleral pathways [8], it can be anticipated that feline eyes with advanced, severe glaucoma pathology impacting these structures would have a weaker response to topical OMDI. Alternatively, EP2 receptor expression in feline eyes may be altered in glaucoma. Although EP2 receptor expression has been documented in normal feline eyes, the effect of glaucoma phenotype or type of glaucoma was not examined. Therefore, further studies are warranted to determine ocular EP2 receptor expression in cats with various types and stages of glaucoma.

## 5 Conclusion

In conclusion, a single topical application of 0.002% OMDI significantly reduces IOP in normal and FCG cats without inducing miosis. These findings suggest that OMDI may represent a viable treatment option for lowering IOP in glaucomatous cats. Further studies are warranted to evaluate the long‐term safety and efficacy and optimal dosing frequency of OMDI in cats with glaucoma.

## Author Contributions

**Kazuya Oikawa:** conceptualization, methodology, investigation, funding acquisition, writing – original draft, visualization, validation, formal analysis, supervision, data curation, project administration. **Julie A. Kiland:** methodology, investigation, validation, formal analysis, supervision, data curation, writing – review and editing, project administration. **Hayden G. Zaluckyj:** investigation, formal analysis, data curation, writing – original draft. **Anjali Rai:** investigation, data curation, writing – review and editing. **Virginia Mathu:** investigation, data curation, writing – review and editing. **Gillian J. McLellan:** conceptualization, methodology, supervision, resources, project administration, writing – review and editing, funding acquisition.

## Funding

This study was supported by the Vision for Animals Foundation (VAF2025‐3); NIH T35 OD011078, T32EY027721 and P30 EY016665, and an unrestricted grant from Research to Prevent Blindness to the Department of Ophthalmology and Visual Sciences, University of Wisconsin‐Madison.

## Disclosure

The authors have not used AI to generate any part of the manuscript.

## Ethics Statement

This study complies with the Guidelines for Ethical Research in Veterinary Ophthalmology (GERVO). All animal procedures were conducted in accordance with the Association for Research in Vision and Ophthalmology (ARVO) Statement on the Use of Animals in Ophthalmic and Vision Research, the NIH Guide for the Care and Use of Laboratory Animals, and in compliance with protocols approved by the Institutional Animal Care and Use Committee at the University of Wisconsin‐Madison.

## Conflicts of Interest

The authors declare no conflicts of interest.

## Data Availability

The data that support the findings of this study are available from the corresponding author upon reasonable request.
